# Supplementary material for: Controlled spatial separation of spins and coherent dynamics in spin-orbit-coupled nanostructures
Source: Nat Commun. 2017 Jul 10;8:15997. doi: 10.1038/ncomms15997 (PMC5508128; doi:10.1038/ncomms15997)
Supplement: Supplementary Information [file ncomms15997-s1.pdf]

Type of file: PDF
Size of file: 0 KB
Title of file for HTML: Supplementary Information
Description: Supplementary Figures, Supplementary Notes and Supplementary References

Supplementary Note 1. Characterization of the spin-orbit-coupled 2DEG and QPCs

Four-terminal magnetotransport measurements are performed to characterize the spin-orbit-coupled two-dimensional electron gas (2DEG) formed in the InGaAs/AlGaAs heterostructure used in this work. Supplementary Figures 1a and 1b show the Hall (ρ_{xy}) and longitudinal (ρ_{xx}) resistivity, respectively, as a function of magnetic field B. At low fields ρ_{xy} increases linearly with B with a slope of $R_{\mathrm{H}} = B/n_{\mathrm{2D}}e$, where n_{2D} is the carrier density and e is the elementary charge. These data are used to obtain the carrier density before and after illumination. Shubnikov-de Haas (SdH) oscillations are observed in $\rho_{xx}(B)$ as a function of B in both cases.

The presence of strong Rashba spin-orbit (SO) coupling lifts the spin degeneracy and leads to a spin splitting of the two-dimensional (2D) subbands. Each spin-polarized subband contains a different density of electrons. The imbalance in carrier density thereby gives rise to a beating pattern in SdH oscillations[1,2]. The beating nodes can be used to calculate the Rashba spin-orbit parameter α using

$$\alpha = \frac{e\hbar}{2k_{\mathrm{F}}m^{*}}\frac{1}{\Delta(1/B)_{\mathrm{beat}}}, \tag{1}$$

where $\Delta(1/B)_{\mathrm{beat}}$ is the beat frequency. A beating pattern is observed in SdH oscillations after illumination (Supplementary Fig. 1c) which gives $\alpha \approx 2.6 \times 10^{-11}$ eVm. The minima in the SdH oscillations are less well defined prior to illumination making the beating nodes harder to identify in this case.

Supplementary Figures 1d and 1e show one-dimensional (1D) conductance quantization in

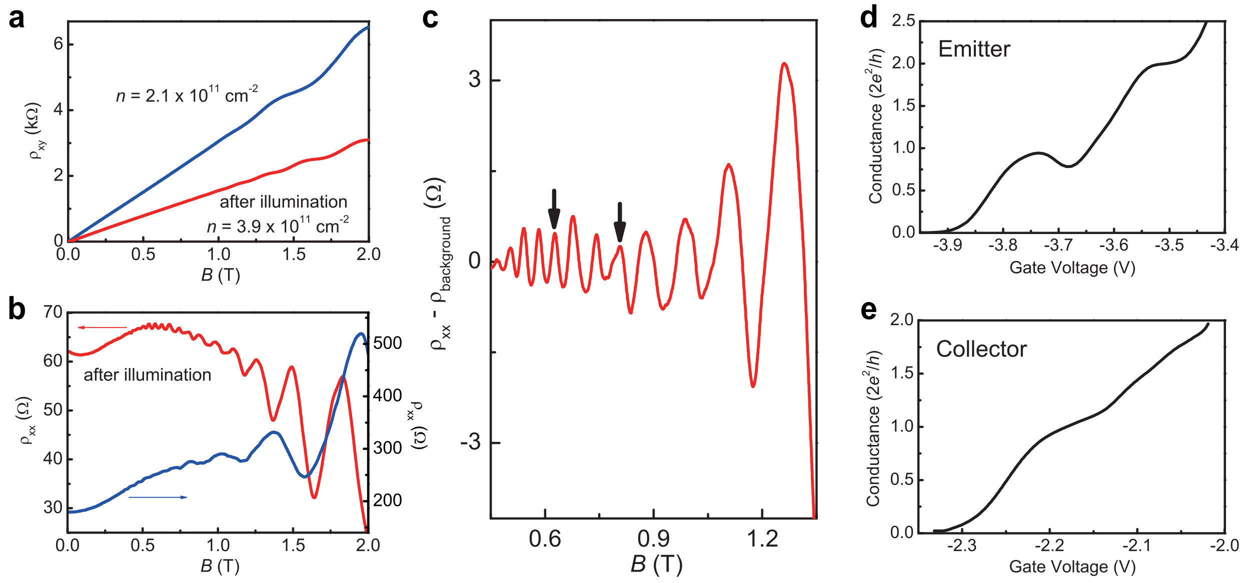

Supplementary Figure 1: **Four-terminal magnetotransport measurements. a**,**b**, Hall (**a**) and longitudinal (**b**) resistivity ρ_{xy} and ρ_{xx}, respectively, as a function of magnetic field B before (blue trace) and after (red trace) illumination. **c**, Longitudinal resistivity after subtraction of the background magnetoresistance for data after illumination. The arrows indicate the position of the beating nodes. **d**,**e**, The conductance as a function of common-mode symmetric gate bias measured for the emitter (**d**) and collector (**e**) QPC.

the emitter and collector quantum point contacts (QPC) in our spin focusing devices. Since the energy of the base of the two 1D spin subbands is the same energy regardless of SO coupling[3,4], the conductance quantization in a QPC with SO coupling appears the same as that without SO coupling. Other approaches, such as the magnetic focusing technique reported in this work, are therefore required to study the effect of SO coupling and its induced spin polarization in a QPC.

Supplementary Note 2. QPC spin filter

The effect of SO coupling is to lift the spin degeneracy in a QPC, shifting the spin up/down 1D subbands horizontally on the k_x axis. In our experimental setup where a small external focusing magnetic field is applied perpendicular to $\mathbf{B}_{\mathrm{R}}^{\mathrm{SO}}$ and parallel to $\mathbf{B}_{\mathrm{L}}^{\mathrm{SO}}$, the spin up/down subbands are shifted vertically and become mixed[5] such that a gap appears near $k_x = 0$, as illustrated in Supplementary Fig. 2a. The QPC acts as a spin injector/detector when operated near threshold, since the spin up and down currents are different.

It is worth noting that the QPC confinement potential also influences the dispersion relation of the two 1D subbands and their spin states[3]. It has also been shown that the spin state for right-moving electrons is opposite to that for left movers[6], in which case the spin filtering action occurs even though the overall spin polarization is zero.

The role of electron-electron interactions may also be important. The electron-electron interactions are significant at low carrier density and can enhance the QPC spin polarization once the symmetry between the two spin species is broken[7–10]. Such strong interactions have recently been demonstrated[11,12] to significantly enlarge the SO-induced spin splitting and result in an overall spin polarization, as manifested in the appearance of a conductance plateau around $0.5 \times 2e^2/h$. This half-integer plateau is not observed in our QPC, which does not rule out the possibility that electron-electron interactions are also enhancing the spin polarization here. At finite temperature it is difficult to evaluate the magnitude of spin polarization from conductance alone, and it is possible that the interaction strength is not large enough for a half-integer plateau to appear with our

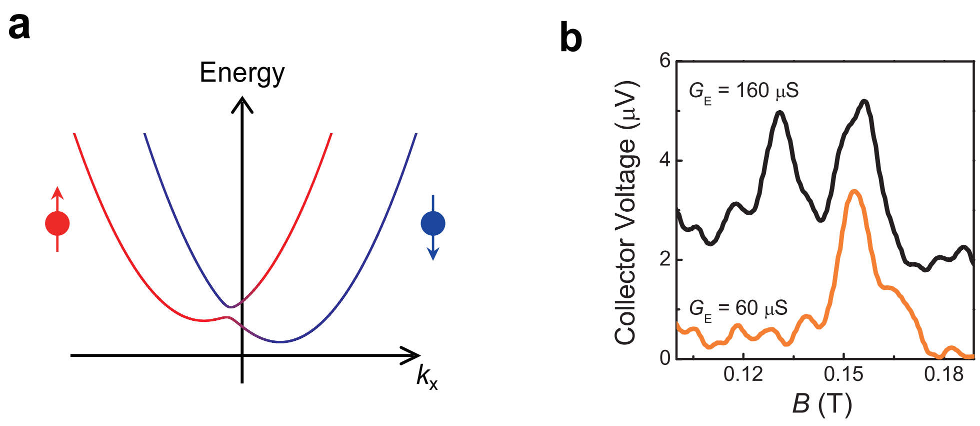

Supplementary Figure 2: **QPC spin filter.** **a**, The dispersion relation of 1D subbands in our experimental setup. Spin filtering occurs when the Fermi level is tuned below the base of the upper energy dispersion relation. In this case electrons moving in either direction are spin polarized. **b**, Transverse magnetic focusing spectrum for emitter conductance $G_{\rm E} = 60$ μS (orange trace) and 160 μS (black trace). The collector conductance is fixed at $G_{\rm C} = 100$ μS. Data are offset vertically for clarity.

confinement potential profile at this temperature.

To determine whether the current injected from the QPC is spin polarized, we directly measure the spin content of the injected current using the focusing technique. This allows us to separate the two spin species and assess the magnitude of the spin-up and spin-down currents. Supplementary Figure 2b shows that both the $B_{\uparrow}$ and $B_{\downarrow}$ focusing peaks are present when the QPC emitter is fixed at $G_{\rm E} = 160$ μS (black trace), indicating that the injected current contains both spin types. In contrast, when the QPC emitter is tuned to 60 μS (orange trace), the $B_{\uparrow}$ peak vanishes and the $B_{\downarrow}$ peak remains. This shows that the injected current only contains the spin-down electrons, and

the QPC now acts as a spin filter. We note that any decoherence or evolution of the spin dynamics of the electron as it travels ballistically do not affect its momentum. Therefore, while the data presented here are able to identify the spin species of the electrons injected from the QPC, they do not shed light on their spin dynamics in the two-dimensional base region.

Supplementary Note 3. Behaviour of higher-order focusing peaks

For the focusing electrons, which have an incident angle of ~ 0 degrees, the reflection from the 2DEG edge is dominated by the spin-flip process[13]. The spin flips during reflection since the direction of $\mathbf{B}_{\mathrm{R}}^{\mathrm{SO}}$ is reversed. It has been shown in theory[14] that due to such spin conversion the second focusing peak (and other even peaks) do not split because the two spatially separated spin branches reunite with each other at the collector, as illustrated in Supplementary Fig. 3 for the second peak. However, splitting is expected for the third focusing peak (and other odd peaks) because these two spin branches separate again after the 2nd reflection process. This is consistent with our experimental results that the splitting appears in the first and third peak but not obvious in the second peak.

Supplementary Note 4. On the physical concept of spin precession in our magnetic focusing setup

A lateral potential asymmetry is introduced across the emitter QPC which is necessary to collapse electron spins into either one of the $\mathbf{B}_{\mathrm{L}}^{\mathrm{SO}} + \mathbf{B}_{\mathrm{R}}^{\mathrm{SO}}$ parallel or antiparallel spin states. Such initial states

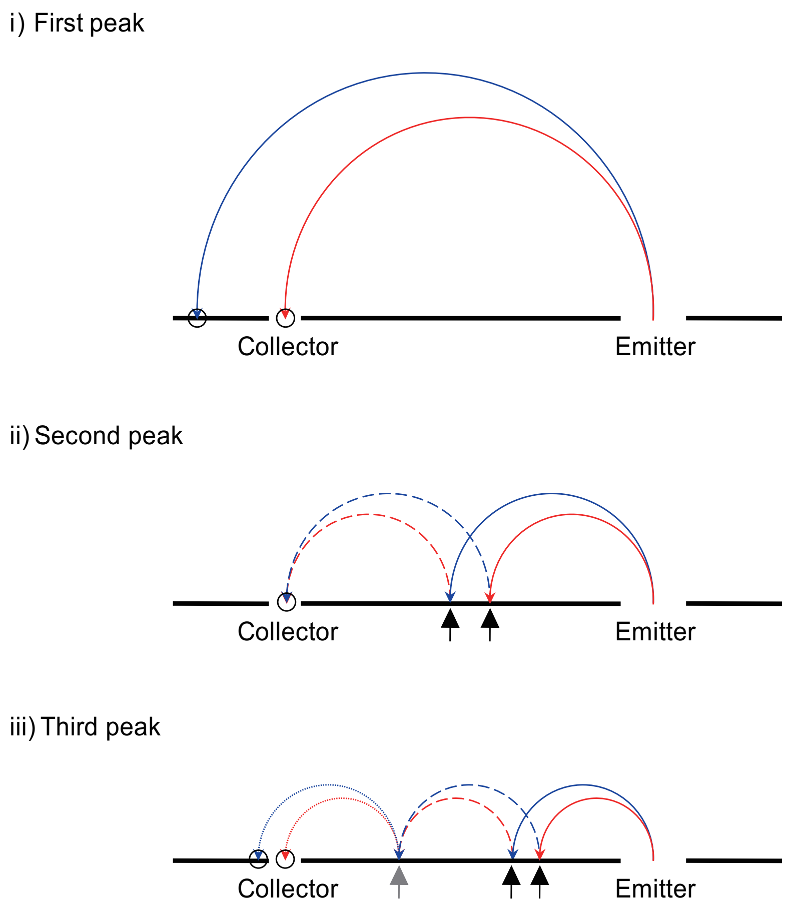

Supplementary Figure 3: **Magnetic focusing schematics.** Cyclotron trajectories of focusing electrons with spins parallel and antiparallel to $\mathbf{B}_{\mathrm{R}}^{\mathrm{SO}}$ (red and blue curves, respectively), which experience no (the first peak), one (the second peak), or two (the third peak) reflections off the 2DEG edge. The arrows indicate the regions where reflection occurs. The circles emphasize how the focusing peak splits when sweeping the transverse magnetic field.

can be written in the $|\pm\rangle_{\mathrm{R}}$ basis:

$$|\psi_{\uparrow\downarrow}(t=0)\rangle = \cos\frac{\theta_{\uparrow\downarrow}}{2}\,|+\rangle_{\mathrm{R}} + \sin\frac{\theta_{\uparrow\downarrow}}{2}e^{i\phi_{\uparrow\downarrow}}\,|-\rangle_{\mathrm{R}}\,, \tag{2}$$

where $|+\rangle_{\mathrm{R}}$ and $|-\rangle_{\mathrm{R}}$ denote the 2DEG eigenstates parallel and antiparallel to $\mathbf{B}_{\mathrm{R}}^{\mathrm{SO}}$. Here the polar and azimuthal angles (θ and ϕ) define the orientation in the Bloch sphere with $\theta_{\downarrow} = \theta_{\uparrow} + \pi$ and $\phi_{\downarrow} = \phi_{\uparrow} + \pi$. When entering into the focusing 2D region, where there is only Rashba SO coupling, these superposition states are no longer stationary and start to evolve under the influence of the rotating $\mathbf{B}_{\mathrm{R}}^{\mathrm{SO}}$. Within the adiabatic approximation, the spinors can be described as a superposition of such adiabatic eigenstates $|\pm\rangle_{\mathrm{R}}$ with conserved $\mathcal{P}(+) = |\,{}_{\mathrm{R}}\langle +|\psi_{\uparrow\downarrow}(t)\rangle\,|^2$ and $\mathcal{P}(-) = |\,{}_{\mathrm{R}}\langle -|\psi_{\uparrow\downarrow}(t)\rangle\,|^2$. Their time evolution can be visualized as precessing spins in the Bloch sphere with a phase given by equation (2) in the main article. For such spin precession to be measured, the collector QPC is tuned to act as a spin projection observable and the spin projection must not be along the axis of $\mathbf{B}_{\mathrm{R}}^{\mathrm{SO}}$, otherwise the probability amplitude would be time independent since $|\pm\rangle_{\mathrm{R}}$ are stationary states. A lateral potential asymmetry is introduced across the collector QPC to achieve this.

Supplementary Note 5. The impact of lateral asymmetry on the collector voltage oscillation

For the spin precession – indicated by the oscillation in the voltage detected across the collector – to be observed, the initial spin preparation (from the emitter) and the eventual spin projection (at the collector) must not align with the Rashba SO effective field $\mathbf{B}_{\mathrm{R}}^{\mathrm{SO}}$. The lateral SO effective field $\mathbf{B}_{\mathrm{L}}^{\mathrm{SO}}$ is therefore required to be introduced into both the emitter and collector QPC to tilt the injected spin away from its rotational axis, $\mathbf{B}_{\mathrm{R}}^{\mathrm{SO}}$, and to analyze the spin precession, respectively. Supplementary

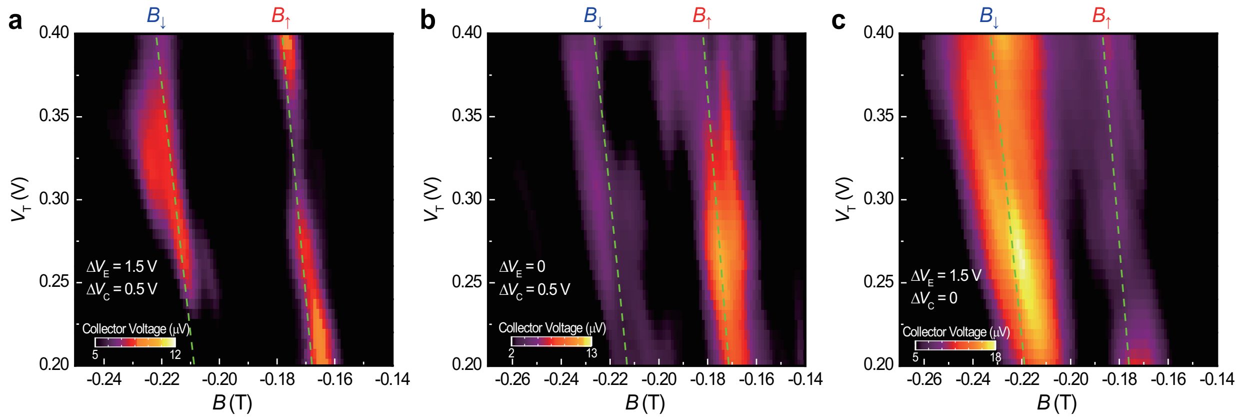

Supplementary Figure 4: **Magnetic spin focusing spectra.** **a**,**b**,**c**, Collector voltage as a function of magnetic field B and top gate voltage V_{T} for device B. The lateral biases applied to the emitter and collector QPC are respectively set at (**a**) $\Delta V_{\mathrm{E}} = 1.5$ V and $\Delta V_{\mathrm{C}} = 0.5$ V; (**b**) $\Delta V_{\mathrm{E}} = 0$ and $\Delta V_{\mathrm{C}} = 0.5$ V; (**c**) $\Delta V_{\mathrm{E}} = 1.5$ V and $\Delta V_{\mathrm{C}} = 0$. The dashed lines show the focusing peak positions calculated using the spin precessional motion.

Figure 4a shows that both the $B_\uparrow$ and $B_\downarrow$ focusing peaks in collector voltage oscillate with V_{T} when both the QPCs are asymmetrically biased with $\Delta V_{\mathrm{E}} = 1.5$ V and $\Delta V_{\mathrm{C}} = 0.5$ V.

When the lateral asymmetry (and hence $\mathbf{B}_{\mathrm{L}}^{\mathrm{SO}}$) is removed from either the emitter or collector QPC, no oscillation in collector voltage is expected. Such behaviour is clearly shown in Supplementary Figs. 4b and 4c, where the focusing peaks associated with $B_\uparrow$ and $B_\downarrow$ do not oscillate if either ΔV_{E} or ΔV_{C} is set to zero.

Supplementary Note 6. Lateral position shift of the QPC

It is possible for the asymmetric biasing of the QPC to shift the spatial position of the conducting channel within the QPC and hence the focusing peak positions. Supplementary Figure 5 shows the position shift of the focusing peaks when the lateral asymmetry of the QPC emitter ΔV_{E} is changed from 1.5 V to -1 V while ΔV_{C} and V_{T} are both fixed to keep the position of the QPC collector, the 2D density, and Rashba parameter α constant. In this case the position shift of the QPC emitter due to the asymmetric biasing is fully responsible for the shift of the focusing peaks and can be easily estimated using equation (1) given in the main article:

$$B_{\uparrow\downarrow} = \frac{2(\sqrt{2m^{*}E_{\mathrm{F}}} \mp m^{*}\alpha/\hbar)}{eL}, \tag{3}$$

which gives $\Delta L \approx -60$ nm.

It may be worth noting that the position shift of the QPC emitter obtained using the method described above is consistent with the value estimated with a simple equation that was commonly used to approximate the position shift due to asymmetric biasing:

$$\Delta L = c\frac{W}{2}\frac{\Delta V_{\mathrm{E}}}{|V_{\mathrm{E1}} + V_{\mathrm{E2}}|}, \tag{4}$$

where W is the width of the split gate and c accounts for the distribution of the electron density in the channel and was found to be $c \sim 0.8$ (Supplementary Ref. 15). The position shift calculated using this equation is -65 nm, very similar to the value obtained using magnetic focusing.

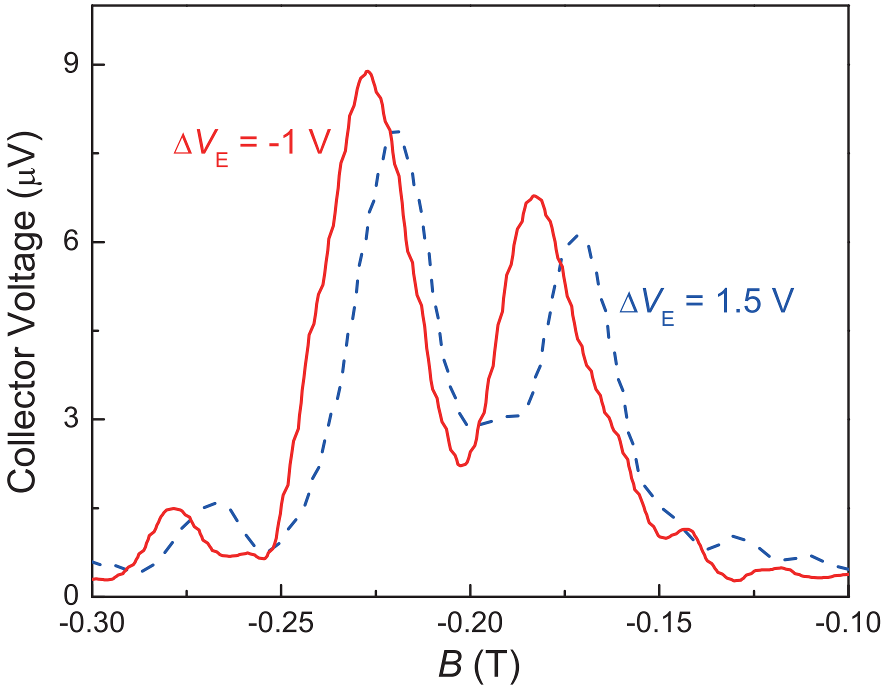

Supplementary Figure 5: **Shift in the focusing peak positions.** Collector voltage as a function of magnetic field B for $\Delta V_{\mathrm{E}} = 1.5$ V (dashed curve) and $\Delta V_{\mathrm{E}} = -1$ V (solid curve). ΔV_{C} and V_{T} are fixed at 0.5 V and 0.3 V, respectively, to keep the position of the QPC collector, the 2D density, and Rashba parameter α constant.

Supplementary References

1. Nitta, J., Akazaki, T., Takayanagi, H. & Enoki, T. Gate control of spin-orbit interaction in an inverted $In_{0.53}Ga_{0.47}As/In_{0.52}Al_{0.48}As$ heterostructure. *Phys. Rev. Lett.* **78**, 1335-1338 (1997).

2. Lo, I. *et al.* Spin splitting in modulation-doped $Al_xGa_{1-x}N/GaN$ heterostructures. *Phys. Rev. B* **65**, 161306 (2002).

3. Moroz, A. V. & Barnes, C. H. W. Effect of the spin-orbit interaction on the band structure and conductance of quasi-one-dimensional systems. *Phys. Rev. B* **60**, 14272-14285 (1999).

4. Quay, C. H. L. *et al.* Observation of a one-dimensional spin-orbit gap in a quantum wire. *Nature Phys.* **6**, 336-339 (2010).

5. Pershin, Y. V., Nesteroff, J. A. & Privman V. Effect of spin-orbit interaction and in-plane magnetic field on the conductance of a quasi-one-dimensional system. *Phys. Rev. B* **69**, 121306 (2004).

6. Governale, M. & Zülicke, U. Spin accumulation in quantum wires with strong Rashba spin-orbit coupling. *Phys. Rev. B* **66**, 073311 (2002).

7. Wang, C.-K. & Berggren, K.-F. Spin splitting of subbands in quasi-one-dimensional electron quantum channels. *Phys. Rev. B* **54**, R14257 (1996).

8. Lassl, A., Schlagheck, P. & Richter, K. Effects of short-range interactions on transport through quantum point contacts: A numerical approach. *Phys. Rev. B* **75**, 045346 (2007).

9. Chen, T. M., Graham, A. C., Pepper, M., Farrer, I. & Ritchie, D. A. Bias-controlled spin polarization in quantum wires. *Appl. Phys. Lett.* **93**, 032102 (2008).

10. Chen, T. M., Pepper, M., Farrer, I., Jones, G. A. C. & Ritchie, D. A. All-electrical injection and detection of a spin-polarized current using 1D conductors. *Phys. Rev. Lett.* **109**, 177202 (2012).

11. Debray, P. *et al.* All-electric quantum point contact spin-polarizer. *Nature Nanotech.* **4**, 759-764 (2009).

12. Wan, J., Cahay, M., Debray, P. & Newrock, R. Possible origin of the 0.5 plateau in the ballistic conductance of quantum point contacts. *Phys. Rev. B* **80**, 155440 (2009).

13. Govorov, A. O., Kalameitsev, A. V. & Dulka, J. P. Spin-dependent transport of electrons in the presence of a smooth lateral potential and spin-orbit interaction. *Phys. Rev. B* **70**, 245310 (2004).

14. Usaj, G. & Balseiro, C. A. Transverse electron focusing in systems with spin-orbit coupling. *Phys. Rev. B* **70**, 041301 (2004).

15. Wakaya, F., Takahara, J., Takaoka, S., Murase, K. & Gamo, K. Confinement potential in an asymmetrically biased quantum point contact. *Jpn. J. Appl. Phys.* **35**, 1329-1332 (1996).
